# Analysis of Hospital Operating Margins and Provision of Safety Net Services

**DOI:** 10.1001/jamanetworkopen.2023.8785

**Published:** 2023-04-18

**Authors:** Lukas K. Gaffney, Kenneth A. Michelson

**Affiliations:** 1 Department of Pediatrics, Boston Medical Center, Boston, Massachusetts; 2 Department of Pediatrics, Boston Children’s Hospital, Boston, Massachusetts; 3 Division of Emergency Medicine, Boston Children’s Hospital, Boston, Massachusetts

## Abstract

**Importance:**

Safety net hospitals (SNH) provide many community services. The cost of providing these services is unknown.

**Objective:**

To determine what safety net criteria are associated with differences in hospital operating margin.

**Design, Setting, and Participants:**

This cross-sectional study of US acute care hospitals from 2017 to 2019 included eligible hospitals identified from US Centers for Medicare & Medicaid Services Cost Reports.

**Exposures:**

Five domains of SNH: undercompensated care measured using the Disproportionate Share Hospital index, uncompensated care, essential community services, neighborhood disadvantage, and sole community hospital and critical access hospital status. Each was categorized as a quintile or binary response. Covariates included hospital ownership, size, teaching status, census region, urbanicity, and wage index.

**Main Outcomes and Measures:**

Operating margin and its association with each safety net criterion was determined using linear regression adjusting for all safety net criteria and covariates.

**Results:**

A total of 4219 hospitals were analyzed, of which 3329 hospitals (78.9%) satisfied at least 1 safety net criterion; 23 hospitals (0.5%) met 4 or all 5 criteria. Among safety net criteria, the highest quintile of undercompensated care (−6.2 percentage point difference compared with lowest quintile; 95% CI, −8.2 to −4.2 percentage points), uncompensated care (−3.4 percentage points; 95% CI, −5.1 to −1.6 percentage points), and neighborhood disadvantage (−3.9 percentage points; 95% CI, −5.7 to −2.1 percentage points) were each associated with a lower operating margin. No association with operating margin was found between critical access or sole community hospital status (0.9 percentage points; 95% CI, −0.8 to 2.7 percentage points) or the highest vs lowest quintile of essential services (0.8 percentage points; 95% CI, −1.2 to 2.7 percentage points). Among essential services, burn, inpatient psychiatry, and primary care services were associated with lower operating margin, while others were either not associated or showed positive association. Fall-off in operating margin by level of uncompensated care was most severe in the highest percentiles of uncompensated care, with the most marked declines among those with the lowest operating margin.

**Conclusions and Relevance:**

In this cross-sectional study of SNH, hospitals in the highest quintiles of undercompensated care, uncompensated care, and neighborhood disadvantage were more financially vulnerable than those not in the top quintile, especially when they met numerous of these criteria. Ensuring targeting of financial support to these hospitals could improve their financial stability.

## Key Points

**Question:**

What is the association between provision of safety-net services and hospital operating margins?

**Findings:**

In this cross-sectional study of 4219 hospitals, higher levels of uncompensated care, low-compensation care, and area socioeconomic disadvantage were associated with lower operating margin, while providing more essential services or being a critical access hospital were not.

**Meaning:**

These results suggest that hospitals with certain safety-net features have increased financial instability and may need additional support.

## Teaser

This cross-sectional study of US acute care hospitals examines the association of several criteria used to designate safety net hospitals with hospital operating margins.

## Introduction

Safety net hospitals (SNH) provide a disproportionate share of services to populations with low access to health care,^1,2^ and are therefore critical to health care delivery nationally. Defining SNH is a challenge. SNH provide many functions and services, but not all SNH fulfill the same role.^3,4^ Traditionally, Medicaid and unreimbursed care proportions have been employed as a proxy for SNH status; however, these are only 2 important characteristics of SNH.^5,6,7^ Other key domains of SNH status include location; provision of essential services such as inpatient psychiatric, substance use, and burn care; patient demographics; hospital finances; and community investment.^4,8,9^

While SNH services benefit communities, they come at a cost. For instance, uncompensated care—the combination of charity care, payments hospitals are unable to collect, and reimbursement shortfalls—totaled $41.3 billion in 2018.^10^ Hospitals with lower profit margins tend to provide higher amounts of charity care, creating a risk for insolvency.^5,11,12^

The disproportionate share hospital (DSH) system was established in 1981 to mitigate these shortfalls.^13^ Because individual states have discretion to set DSH payment distribution policy, there is significant heterogeneity in determining which hospitals receive DSH payments.^14^ Resultingly, these appropriations have not been found to be associated with any SNH factors, such as uncompensated care or provision of essential services.^14,15,16^ Many hospitals rely on these payments for fiscal stability, and planned reductions to federal DSH funding over the coming years may further complicate these distribution patterns and financially strain SNH.^12^

The marginal cost of providing safety net services is uncertain. Understanding these costs could inform targeted support, such as DSH appropriations. In turn, improved policies could decrease the elevated risk of closure for hospitals that provide care to underinsured and uninsured populations.^17,18^ Our objective was to quantify the operational costs of providing a range of SNH services. We hypothesized that hospitals with high levels of uncompensated and undercompensated care would be associated with lower operating margins.

## Methods

### Design, Setting, and Participants

We conducted a cross-sectional study of US acute care hospitals from 2017 through 2019, evaluating the association of safety net hospital status with operating margin. We excluded hospitals missing data on essential services, uncompensated care, operating margin, or wage index. We followed the Strengthening the Reporting of Observational Studies in Epidemiology (STROBE) reporting guideline for cross-sectional studies. Informed consent was not required because this study did not meet institutional review board criteria for human subjects.

### Data Sources

Data were obtained from the US Centers for Medicare & Medicaid Services (CMS) Cost Reports 2017-2019.^19^ Our approach to developing an analytic data set from the cost reports is described in the Supplement (eMethods in Supplement 1). Presence of essential community services data was drawn from the American Hospital Association (AHA) Annual Survey.

### Outcomes

The primary outcome was the operating margin, defined as net income from patient care (operating revenue minus operating expenses) divided by revenue from patient care.^20^ Operating margin was preferred over total margin as total margin incorporates various activities unrelated to patient care that are volatile and may be reported differently across hospitals.^21,22^ Operating margin was winsorized at the 2.5th and 97.5th percentiles (eg, costs lower than the 2.5th percentile were set to the 2.5th percentile) to account for outliers. A secondary outcome was financial stability, defined as a positive operating margin.

### SNH Definitions

We measured SNH status in 5 domains^4^: undercompensated care, uncompensated care, essential community services, neighborhood disadvantage, and sole community hospital (SCH) or critical access hospital (CAH) status. Undercompensated care was measured using the Medicare DSH index, which represents the sum of a hospital’s proportions of patients with Medicare Supplemental Security Income among all Medicare patients plus those with Medicaid insurance among all non-Medicare patients.^23^ Hospitals must exceed a DSH index of 0.15 to qualify for DSH payment, and they are legally required to report their DSH index only if they receive DSH payments.^24^ For cases of missing DSH index, we assigned a value of 0, as nonreporting hospitals are likely to have a very low index based on their nonreceipt of DSH payments.

Uncompensated care was determined as the ratio of charity care and bad debt to hospital bed count.^10^ Because the amounts reported reflect charges and not costs, we multiplied uncompensated care by winsorized hospital-specific cost-to-charge ratios.^25^ The final amount was again winsorized at the 2.5th and 97.5th percentile to account for uncompensated care per bed outliers.

Essential community services were defined by the Medicaid and Children’s Health Insurance Program Payment and Access Commission and included: burn, dental, HIV and AIDS, neonatal intensive care, obstetrics and gynecology, primary care, substance use treatment, trauma, graduate medical education, and inpatient psychiatry.^14^ Each hospital received a score from zero to 10 based on the total number of these services that it provided.

Area disadvantage was defined by Area Deprivation Index (ADI), a measure that ranks neighborhoods by relative socioeconomic disadvantage and includes factors such as income, education, employment, and housing quality.^26,27^ In this study, ADI was assigned based on a hospital’s census block group location. When ADI was not available for a given block group, the next nearest block group was used based on block group centroids.

Hospitals designated under specific Medicare rules as CAH or SCH were considered to serve safety net functions due to their geographic isolation.^7,28,29^ Each SNH criterion was categorized by quintile or as a binary response for CAH or SCH status.

### Covariates and Potential Confounders

Covariates and confounders were each selected because of their known association with safety net features and our hypothesized association with operating margin.^3,22^ These included ownership type (for profit, governmental, nonprofit), size (fewer than 100 beds, 100 to 299 beds, 300 beds or more), teaching status, census region (Northeast, Midwest, South, West), urbanicity (urban or rural) as defined by the Office of Management and Budget,^30^ and wage index, obtained from CMS. Wage index was determined at the level of core-based statistical area (CBSA), and all regions outside of CBSAs were grouped as rural areas by each individual state (eg, rural Alabama).^31^

### Statistical Analysis

We reported demographic characteristics of hospitals by the number of SNH criteria met. For demographic reporting, hospitals were categorized as meeting SNH criteria if they were in the top quintile for the particular measure, or if they had CAH or SCH status.^32^

Associations between operating margin and each SNH criterion were determined using linear regression with operating margin as the dependent variable. First, we created a base model including only hospital characteristics that we theorized a priori could confound the association between SNH services and operating margin: ownership, teaching status, census region, urbanicity, and wage index. We next created separate models for each SNH criterion by adding the criterion to the base model. Finally, to isolate the independent association of each SNH criterion relative to the others, we created a full model as the base model plus all 5 SNH criteria. As a related evaluation of how SNH status might impact financial stability, we also recreated the full model using logistic regression with financial stability as the outcome.

Different essential community services could affect operating margins in substantially different ways. To examine this possibility, we developed models with each of the 10 essential community services individually and then combined, both including all possible confounders.

We performed several sensitivity analyses to evaluate the effect of imputing DSH indices as zero when missing. These included setting missing DSH index to the lowest reported in the state, setting them to the state-specific 25th percentile, and repeating the model restricting to only those hospitals that reported a DSH index.

After analyzing our initial results, we added post hoc exploratory analyses, which we regarded as hypothesis-generating. First, we examined the operating margin of hospitals that were in the highest quintile of numerous SNH criteria. We then explored the average operating margin by ventiles to probe for possible dose-dependent or threshold effects.

Bivariable comparisons used a χ^2^ tests or rank sum tests as appropriate. All analyses were performed in R version 4.1.2 (R Foundation). Statistical significance was defined as *P* < .05 in 2-sided tests.

## Results

### Hospital Characteristics

Among 4453 US acute care hospitals, we excluded 94 for missing essential services, 78 for missing operating margin, 59 for missing uncompensated care, and 3 for missing wage index. We analyzed 4219 (94.7%) hospitals (Table 1). Excluded hospitals were more likely to be for profit, located in the West, urban, and have graduate medical education than the included hospitals.

**Table 1.** Demographic Features of the Cohort Stratified by Number of Safety Net Hospital Criteria

Characteristic	Hospitals, No. (%)					
	Overall (N = 4219)	Safety net hospital criteria satisfied^a^				
		0 (n = 890)	1 (n = 1616)	2 (n = 1330)	3 (n = 360)	4-5 (n = 23)
Ownership						
For profit	671 (15.9)	243 (27.3)	269 (16.6)	121 (9.1)	38 (10.6)	0
Governmental	950 (22.5)	81 (9.1)	315 (19.5)	402 (30.2)	138 (38.3)	14 (60.9)
Nonprofit	2598 (61.6)	566 (63.6)	1032 (63.9)	807 (60.7)	184 (51.1)	9 (39.1)
Bed count						
<100	2289 (54.3)	366 (41.1)	856 (53.0)	839 (63.1)	216 (60.0)	12 (52.2)
100-299	1270 (30.1)	430 (48.3)	516 (31.9)	254 (19.1)	66 (18.3)	4 (17.4)
≥300	660 (15.6)	94 (10.6)	244 (15.1)	237 (17.8)	78 (21.7)	7 (30.4)
GME	1151 (27.3)	210 (23.6)	438 (27.1)	366 (27.5)	128 (35.6)	9 (39.1)
Census region						
Midwest	1301 (30.8)	208 (23.4)	543 (33.6)	458 (34.4)	90 (25.0)	2 (8.7)
Northeast	533 (12.6)	139 (15.6)	218 (13.5)	134 (10.1)	41 (11.4)	1 (4.3)
South	1551 (36.8)	407 (45.7)	505 (31.2)	441 (33.2)	179 (49.7)	19 (82.6)
West	834 (19.8)	136 (15.3)	350 (21.7)	297 (22.3)	50 (13.9)	1 (4.3)
Urban	2078 (49.3)	697 (78.3)	800 (49.5)	441 (33.2)	129 (35.8)	11 (47.8)
DSH index, median (IQR)	0.22 (0-0.33)	0.21 (0.16-0.28)	0.23 (0-0.32)	0.16 (0-0.38)	0.23 (0-0.46)	0.43 (0.38-0.52)
Uncompensated care, median (IQR), bed/y	12 734 (6849-24 031)	8908 (5232-14 662)	11 184 (6324-18 382)	19 327 (9221-36 120)	33 674 (16 384-56 413)	37 128 (29 813-47 368)
SCH	463 (11.0)	0	162 (10.0)	213 (16.0)	75 (20.8)	13 (56.5)
CAH	1287 (30.5)	0	492 (30.4)	628 (47.2)	166 (46.1)	1 (4.3)
ADI, median (IQR)	64 (40-81)	49 (29-66)	62 (40-77)	71 (47-87)	88 (74-94)	91 (88-95)
Essential services						
Burn	391 (9.3)	20 (2.2)	110 (6.8)	174 (13.1)	80 (22.2)	7 (30.4)
Dental	1294 (30.7)	206 (23.1)	479 (29.6)	436 (32.8)	161 (44.7)	12 (52.2)
HIV/AIDS	1324 (31.4)	222 (24.9)	519 (32.1)	423 (31.8)	146 (40.6)	14 (60.9)
NICU	1300 (30.8)	221 (24.8)	497 (30.8)	417 (31.4)	150 (41.7)	15 (65.2)
Obstetrics	3034 (71.9)	668 (75.1)	1168 (72.3)	933 (70.2)	242 (67.2)	23 (100.0)
Primary care	2651 (62.8)	393 (44.2)	1023 (63.3)	944 (71.0)	274 (76.1)	17 (73.9)
Substance use	735 (17.4)	62 (7.0)	268 (16.6)	288 (21.7)	105 (29.2)	12 (52.2)
Trauma	2271 (53.8)	327 (36.7)	855 (52.9)	818 (61.5)	251 (69.7)	20 (87.0)
GME	1717 (40.7)	368 (41.3)	649 (40.2)	520 (39.1)	169 (46.9)	11 (47.8)
Inpatient psychiatric	1710 (40.5)	275 (30.9)	639 (39.5)	587 (44.1)	194 (53.9)	15 (65.2)

Abbreviations: ADI, Area Deprivation Index; CAH, critical access hospital; DSH, disproportionate share hospital; GME, graduate medical education; NICU, neonatal intensive care unit; SCH, sole community hospital.

^a^ Satisfying a criterion was defined as being in the top quintile for that criterion or qualifying for CAH or SCH designation.

Of analyzed hospitals, 3329 (78.9%) satisfied at least 1 SNH criterion, and 23 hospitals (0.5%) met 4 or all 5 criteria. There was generally a lower percentage of for-profit hospitals and higher percentage of governmental hospitals as the number of SNH criteria increased. Uncompensated care, DSH index, ADI, and essential community services percentages all increased as more SNH criteria were satisfied. Among hospitals meeting 4 or 5 criteria, SCH (13 hospitals [56.5%]) were more common than CAH (1 hospital [4.3%]).

### Model Results

In the base model, each potential confounder was found to be significantly associated with operating margin except wage index (operating margin change of −3.4 percentage points; 95% CI, −7.1 to 0.3 percentage points). Increasing bed count was associated with higher operating margin: compared with less than 100 beds, having 100 to 299 beds (5.4 percentage points; 95% CI, 4.0 to 6.8 percentage points) or 300 or more beds (7.8 percentage points; 95% CI, 6.0 to 9.6 percentage points) was positively associated with operating margin. Compared with the Midwest region, the Northeast (−7.2 percentage points; 95% CI, −8.8 to −5.5 percentage points) and South (−4.5 percentage points; 95% CI, −5.7 to −3.3 percentage points) were each associated with lower operating income, but the West was not (−0.2 percentage points; 95% CI, −1.8 to 1.3 percentage points). Urban location was associated with a higher operating margin compared to rural areas (2.1 percentage points; 95% CI, 0.9 to 3.4 percentage points). Nonprofit hospitals (−3.7 percentage points; 95% CI, −5.0 to −2.3 percentage points) and government hospitals (−14.0 percentage points; 95% CI, −15.6 to −12.3 percentage points) were associated with lower operating income compared with for-profit hospitals. Having an Accreditation Council for Graduate Medical Education residency program was associated with lower operating margin (−2.6 percentage points; 95% CI, −4.0 to −1.2 percentage points).

Among SNH criteria, uncompensated care, undercompensated care, and area disadvantage were each associated with a lower operating margin (Table 2). In the full model, the top quintiles of ADI (−3.9 percentage points; 95% CI, −5.7 to −2.1 percentage points), DSH index (−6.2 percentage points; 95% CI, −8.2 to −4.2 percentage points), and uncompensated care (−3.4 percentage points; 95% CI, −5.1 to −1.6 percentage points) were independently associated with a lower operating margin. Compared with the lowest quintile of essential services, the second (2.7 percentage points; 95% CI, 1.3 to 4.2 percentage points), third (2.9 percentage points; 95% CI, 1.3 to 4.5 percentage points), and fourth (2.0 percentage points; 95% CI, 0.5 to 3.6 percentage points) quintiles of essential services were associated with higher operating margin, but no association was found with the top quintile (0.8 percentage points; 95% CI, −1.2 to 2.7 percentage points). CAH or SCH status was not significantly associated with operating margin (0.9 percentage points; 95% CI, −0.8 to 2.7 percentage points).

**Table 2.** Change in Operating Margin (OM) by Safety Net Hospital Criterion

Quintile^a^	Change in OM, percentage points (95% CI)		Financial stability OR (95% CI)
	Base	Full	
ADI			
Q1 (1-34)	[Reference]	[Reference]	1 [Reference]
Q2 (35-55)	0.3 (−1.2 to 1.9)	0.2 (−1.4 to 1.7)	0.99 (0.79 to 1.24)
Q3 (56-71)	0.2 (−1.5 to 1.9)	0.3 (−1.4 to 2.0)	1.03 (0.81 to 1.32)
Q4 (72-84)	−2.0 (−3.8 to −0.2)^b^	−1.7 (−3.5 to 0.1)	1.24 (0.96 to 1.60)
Q5 (85-100)	−4.4 (−6.2 to −2.6)^c^	−3.9 (−5.7 to −2.1)^c^	1.75 (1.35 to 2.28)^c^
CAH/SCH	0.5 (−0.9 to 2.0)	0.9 (−0.8 to 2.7)	0.96 (0.75 to 1.24)
DSH			
Q1 (0)	[Reference]	[Reference]	1 [Reference]
Q2 (0-0.2)	1.4 (−0.6 to 3.3)	−0.1 (−2.4 to 2.1)	0.64 (0.46 to 0.89)^b^
Q3 (0.2-0.3)	1.6 (0.0 to 3.2)^b^	0.0 (−1.9 to 1.9)	0.70 (0.53 to 0.93)^b^
Q4 (0.3-0.4)	1.2 (−0.4 to 2.8)	−0.2 (−2.1 to 1.7)	0.79 (0.59 to 1.04)
Q5 (0.4-1.2)	−5.2 (−7.0 to −3.5)^c^	−6.2 (−8.2 to −4.2)^c^	1.57 (1.16 to 2.12)^b^
Essential services			
Q1 (0-2)	[Reference]	[Reference]	1 [Reference]
Q2 (3)	2.6 (1.1 to 4.0)^c^	2.7 (1.3 to 4.2)^c^	0.90 (0.73 to 1.12)
Q3 (4)	3.1 (1.5 to 4.7)^c^	2.9 (1.3 to 4.5)^c^	0.97 (0.77 to 1.23)
Q4 (5-6)	2.0 (0.5 to 3.5)^b^	2.0 (0.5 to 3.6)^b^	0.88 (0.70 to 1.11)
Q5 (7-10)	−0.1 (−2.0 to 1.9)	0.8 (−1.2 to 2.7)	1.10 (0.83 to 1.46)
Uncompensated care			
Q1 (1979-5949)	[Reference]	[Reference]	1 [Reference]
Q2 (5957-10 261)	2.3 (0.7 to 3.8)^b^	1.7 (0.3 to 3.2)^b^	0.83 (0.67 to 1.03)
Q3 (10 262-16 455)	1.4 (−0.1 to 3.0)	0.6 (−1.0 to 2.1)	0.87 (0.69 to 1.09)
Q4 (16 460-27 768)	1.8 (0.1 to 3.4)^b^	0.7 (−0.9 to 2.4)	0.83 (0.65 to 1.05)
Q5 (27 770-91 670)	−2.1 (−3.8 to −0.4)^b^	−3.4 (−5.1 to −1.6)^c^	1.10 (0.84 to 1.42)

Abbreviations: ADI, Area Deprivation Index; CAH, critical access hospital; DSH, disproportionate share hospital; OR, odds ratio; SCH, sole community hospital.

^a^ Each quintile for each safety net hospital criterion was compared with the bottom quintile (or absence of CAH or SCH status) after adjusting for ownership, size, teaching status, census region, urbanicity, and wage index (base model) or after adjustment including all other safety net criteria (full models). A logistic regression model using the outcome of financial stability (defined as positive or negative operating margin) is also presented using the same full model covariates.

^b^ *P* < .05.

^c^ *P* < .001.

Overall, 2605 (61.7%) of hospitals had financial instability. Hospitals in the top quintile of uncompensated care (632 hospitals [74.9%]), DSH index (556 hospitals [66.0%]), and ADI (615 hospitals [73.2%]) had higher levels of financial instability. Hospitals in the top quintile of essential services had lower financial instability (415 hospitals [58.5%]). Among hospitals with CAH or SCH status, 1268 hospitals (72.5%) had financial instability. Governmental (adjusted odds ratio [aOR], 6.15; 95% CI, 4.71-8.04) and not-for-profit (aOR, 1.80; 95% CI, 1.47-2.21) hospitals, hospitals with fewer than 100 beds (aOR, 1.54; 95% CI, 1.24-1.91), and hospitals in the Northeast (aOR, 3.29; 95% CI, 2.56-4.24) were associated with financial instability relative to their comparators. Similar to the full model, the top quintiles of area deprivation (aOR, 1.75; 95% CI, 1.35-2.28) and undercompensated care (aOR, 1.57; 95% CI, 1.16-2.12) were associated with financial instability compared with the bottom quintiles.

Among specific essential services, burn services (operating margin increase: −3.0 percentage points; 95% CI, −4.9 to −1.0 percentage points), inpatient psychiatry services (−2.6 percentage points; 95% CI, −3.8 to −1.4 percentage points), and primary care (−1.6 percentage points; 95% CI, −2.6 to −0.5 percentage points) were each significantly associated with lower operating margin. By contrast, obstetric care (3.8 percentage points; 95% CI, 2.6 to 5.0 percentage points), neonatal care (2.0 percentage points; 95% CI, 0.7 to 3.5 percentage points), dental care (1.6 percentage points; 95% CI, 0.4 to 2.7 percentage points), and having residents (2.6 percentage points; 95% CI, 1.1 to 4.0 percentage points) were each significantly associated with a higher operating margin. Trauma (0 percentage points; 95% CI, −1.0 to 1.0 percentage points), substance use treatment (−0.2 percentage points; 95% CI, −1.6 to 1.3 percentage points), and HIV or AIDS (−0.8 percentage points; 95% CI, −2.0 to 0.5 percentage points) services were not associated with operating margin.

### Sensitivity Analyses

When values of nonreported DSH index were reassigned, we observed similar results to the main analysis (Table 3). In the adjusted model, the change in operating margin between the highest and lowest quintiles did not significantly vary when assigning missing DSH indices using the state-specific 25th percentile value (−4.7; 95% CI, −6.3 to −3.3), lowest reported value by state (−5.9; 95% CI, −7.8 to −4.0), or limited to those with reported DSH index (−7.6; 95% CI, −9.5 to −5.7). When limited to hospitals with reported DSH, the fourth quintile was also independently associated with a lower operating margin (−2.0; 95% CI, −3.8 to −0.1). Unchanged associations were observed among other variables.

**Table 3.** Results of Sensitivity Analysis^a^

Quintile	Original^b^	25th Percentile^c^	Lowest^d^	Reported^e^
**Base model**				
Q1 (0)	[Reference]	[Reference]	[Reference]	[Reference]
Q2 (0-0.2)	1.4 (−0.6 to 3.3)	2.9 (1.5 to 4.4)	1.6 (0.1 to 3.1)	0.2 (−1.6 to 1.9)
Q3 (0.2-0.3)	1.6 (0.0 to 3.2)	2.0 (0.5 to 3.5)	1.1 (−0.5 to 2.8)	−0.2 (−2.0 to 1.6)
Q4 (0.3-0.4)	1.2 (−0.4 to 2.8)	1.4 (−0.1 to 2.9)	1.2 (−0.5 to 2.9)	−2.4 (−4.2 to −0.5)
Q5 (0.4-1.2)	−5.2 (−7.0 to −3.5)	−4.9 (−6.5 to −3.3)	−5.3 (−7.1 to −3.5)	−8.7 (−10.6 to −6.8)
**Full model**				
Q1 (0)	[Reference]	[Reference]	[Reference]	[Reference]
Q2 (0-0.2)	−0.1 (−2.4 to 2.1)	2.9 (1.4 to 4.3)	0.9 (−0.6 to 2.5)	0.4 (−1.4 to 2.2)
Q3 (0.2-0.3)	0.0 (−1.9 to 1.9)	1.8 (0.3 to 3.3)	−0.1 (−1.9 to 1.7)	0.1 (−1.7 to 2.0)
Q4 (0.3-0.4)	−0.2 (−2.1 to 1.7)	1.2 (−0.3 to 2.8)	0.0 (−1.8 to 1.9)	−2.0 (−3.8 to −0.1)
Q5 (0.4-1.2)	−6.2 (−8.2 to −4.2)	−4.7 (−6.3 to −3.0)	−5.9 (−7.8 to −4.0)	−7.6 (−9.5 to −5.7)

^a^ Each quintile of DSH was compared with the bottom quintile after adjusting for ownership, size, teaching status, census region, urbanicity, and wage index (base model) or after adjustment including all other safety net criteria (full models).

^b^ Hospitals with unreported disproportionate share hospital (DSH) index were assigned a value of 0.

^c^ Hospitals with unreported DSH index were assigned the 25th percentile reported value of their state.

^d^ Hospitals with unreported DSH index were assigned the lowest reported value of their state.

^e^ Analysis limited to only hospitals with reported DSH index.

### Exploratory Analysis

When exploring additive effects of multiple SNH criteria, hospitals that were in the highest quintile for both DSH index and uncompensated care had the lowest median (IQR) operating margin (−0.22 [−0.38 to −0.08]). Hospitals in the highest quintile for DSH, uncompensated care, and ADI (−0.09 [−0.49 to −0.02]) and hospitals in the highest quintile for ADI and uncompensated care (−0.10 [−0.28 to −0.03]) had the next lowest median operating margins. Hospitals meeting only 1 of these criteria in the highest quintile or both ADI and DSH in the highest quintile had median operating margins in the −0.04 to −0.06 range. Hospitals not meeting any of these 3 criteria in the highest quintile had the highest operating margin of −0.01. When examining uncompensated care specifically, the fall-off in operating margin as the percentile of uncompensated care increased was more marked among hospitals with lower operating margins as the percentile of uncompensated care increased (Figure).

**Figure.**
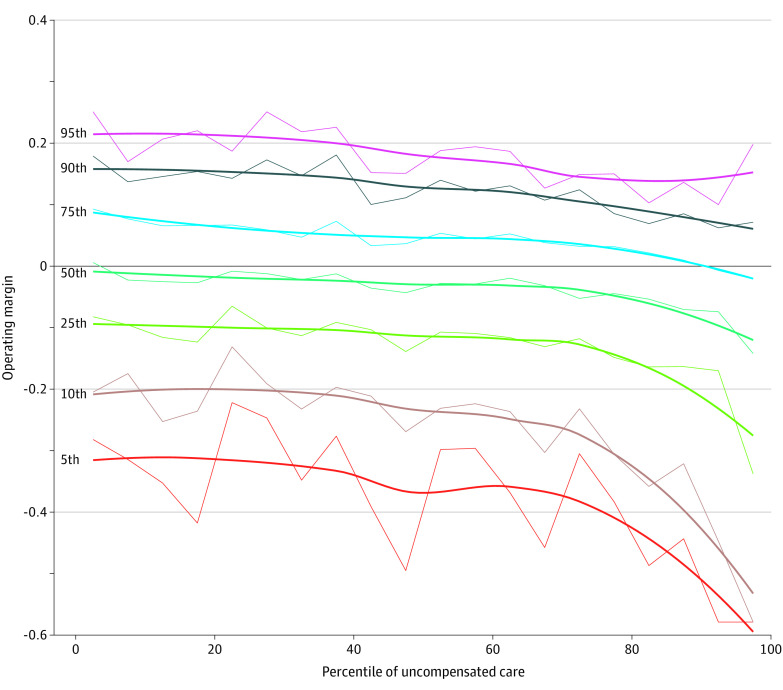
Hospital Operating Margin by Percentile at Corresponding Levels of Uncompensated Care Thin lines are raw data. Thick lines are smoothed trends using quadratic LOESS regression with the alpha parameter set to 0.75.

## Discussion

In US acute care hospitals, the highest quintiles of uncompensated care, undercompensated care, and area disadvantage were associated with lower operating margins and increased financial instability. Neither CAH and SCH status nor higher numbers of essential community services were associated with operating margin. Among essential services, burn, substance use, inpatient psychiatry, and primary care services were associated with lower operating margin, while others were either not associated or showed positive association. Hospitals with both the highest levels of uncompensated care and the highest levels of undercompensated care or area disadvantage demonstrated the lowest median operating margin. Overall, our findings indicated that uncompensated care, DSH index, and ADI are associated with a lower operating margin in a non–dose dependent fashion.

That uncompensated care would negatively impact operating margin is unsurprising, as providing patient care without revenue should theoretically reduce income without affecting expenses. Similarly, a higher DSH index represents more Medicare and Medicaid patients, whose care is reimbursed at a lower rate than private insurance.^33^ High ADI areas have been shown to have greater percentages of uninsured patients. Additionally, patients from lower socioeconomic status areas have higher readmission rates, increasing penalties and decreasing bonuses from the Hospital Readmission Rates Program.^34,35^ Accounting for ADI-related risk factors would cut Hospital Readmissions Rates Program penalties for more than half of SNH, emphasizing the financial impact for hospitals with high numbers of these patients.^34^

For each of the SNH criteria, the association with operating margin applied only to the top quintile. This result may suggest that hospitals have an ability to offset a certain amount of this care with reimbursement from private payers, but that once a certain threshold has been passed these socioeconomic and care provision variables will factor into hospital margins. In addition, for uncompensated and undercompensated care, the top quintile comprised approximately 75% of the range of the median operating margins, indicating that a minority of hospitals provide the bulk of these types of SNH care. When examining different operating margin percentiles by level of uncompensated care, the hospitals with the lowest operating margins demonstrated the steepest fall off in this top quintile. Other factors—such as ownership type—may influence this trend. For instance, nonprofit and government hospitals were less financially solvent than for-profit hospitals, concordant with the idea that for-profit hospitals are less likely to have a need for safety net funds. It remains unclear why some hospitals are more stable despite high levels of uncompensated care, and further exploration of these dynamics could help guide policy to better support those who are struggling.

The number of essential services and CAH or SCH status were not associated with operating margin in a dose-dependent manner. Middle quintiles of essential services were associated with an increase in operating margin. This may be because certain essential services are profitable while others are not. More hospitals provide obstetric care than burn or dental services, and provision of more profitable services may drive this difference. As a result of higher reimbursement levels from their designations, CAH and SCH realize 25% greater payment, on average, than they would without the designations.^17,36^ The lack of correlation with operating margin may suggest effectiveness in that intervention.^28,29,36^

Financial insolvency is a driving factor contributing to hospital closure.^17,18,37^ Because uncompensated care, undercompensated care, and area disadvantage are associated with financial instability, SNH are likely at higher risk of closure. Given the role these hospitals serve for their communities, the implications of such closures would include outsized impacts on patient care among disenfranchised and systematically marginalized communities.^16,17^ Even if they remain in operation, hospitals with poor financial health have poorer safety and quality outcomes.^38^ Further work examining the role of economic pressures such as competition with other hospitals will help further tease out the balance between internal and external factors in closures and patient outcomes.

Federal and state financial assistance to SNH fulfills a vital public health service. Most current federal and state assistance is provided through the DSH program, but this program has not been found to be aligned with any stated target metrics.^4,13,16,39,40^ Our results support working toward an approach that incorporates quantitative SNH measures to better capture both financial risk and community support. Furthermore, moving away from traditional cut-offs to scaled models could better capture the totality of care provided and balance the varied levels of contributions in individual domains across hospitals.^4^ Facing rising health costs,^39^ ongoing revenue losses from COVID-19,^41^ and upcoming reductions in federal funding,^13^ SNH require evidence- and patient-based support.

## Limitations

This study had several limitations. First, DSH index measures were missing for 32% of hospitals in the study. While we presume these values are low (hospitals need to meet a threshold to receive any DSH funds, and all hospitals that receive such funds are required to report their DSH index), we cannot know their exact value. However, our sensitivity analysis suggests that assigning a reasonable range of values or excluding these hospitals generates comparable results. Second, the CMS cost reports can be subject to varying approaches to financial accounting.^14,21^ We chose operating income as our main outcome to isolate the financial considerations of patient care. Other sources of income are vital for many hospitals to offset operational losses and factor into overall institutional health,^22^ although we would argue that the patient care mission of a hospital should not need to be funded by nonoperating income.^22^ A hospital’s ADI may also not capture the complete socioeconomic picture of its catchment area.

## Conclusion

In this cross-sectional study, provision of the highest levels of uncompensated and undercompensated care was associated with substantially lower operating margins. Targeting funds to hospitals with the greatest safety net mission has the potential to improve the financial health of institutions charged with providing the most care to marginalized groups.

## Notes

Supplement 1.
eMethods

Supplement 2.
Data Sharing Statement
