# Supplementary material for: Analysis of Hospital Operating Margins and Provision of Safety Net Services
Source: JAMA Netw Open. 2023 Apr 18;6(4):e238785. doi: 10.1001/jamanetworkopen.2023.8785 (PMC10114034; doi:10.1001/jamanetworkopen.2023.8785)
Supplement: Supplement 1. — eMethods [file jamanetwopen-e238785-s001.pdf]

# Supplemental Online Content

Gaffney LK, Michelson KA. Analysis of hospital operating margins and provision of safety-net services. *JAMA Netw Open.* 2023;6(4):e238785. doi:10.1001/jamanetworkopen.2023.8785

**eMethods.**

This supplemental material has been provided by the authors to give readers additional information about their work.

© 2023 Gaffney LK et al. *JAMA Network Open.*

## eMethods

For the main analysis we used data from the CMS Cost Reports. Hospitals typically submit reports on variable schedules including partial or full years. As such, hospitals had a variable number of reports during the study period. To generate a single final value for each hospital and each variable, we used the modal non-missing value weighted by time. For instance, if a hospital were classified as privately owned for 200 days and publicly owned for 100 days, it was assigned a value of private ownership. For ratios and proportions (such as cost-to-charge ratio), we used the modal non-missing value weighted by time. For dollar amounts, we used the sum of non-missing values. Values were compared to other data sources to establish consistency and validity.[1]

For Disproportionate Share Index (DSH), we reassigned values when outside the possible range. The theoretical maximum value for DSH index is 2, and in our data set there were 2 hospitals with values greater than 2. In both instances there appeared to be errors in decimal placement, and the values were divided by 100.

References:
1. Sacarny A. Data | Adam Sacarny. http://sacarny.com/data/. Accessed May 10, 2022.

© 2023 Gaffney LK et al. *JAMA Network Open.*
