# Supplementary material for: Analysis of Hospital Operating Margins and Provision of Safety Net Services
Source: JAMA Netw Open. 2023 Apr 18;6(4):e238785. doi: 10.1001/jamanetworkopen.2023.8785 (PMC10114034; doi:10.1001/jamanetworkopen.2023.8785)
Supplement: Supplement 2. — Data Sharing Statement [file jamanetwopen-e238785-s002.pdf]

Data Sharing Statement

Gaffney. Analysis of Hospital Operating Margins and Provision of Safety Net Services. *JAMA Netw Open*. Published April 18, 2023. doi:10.1001/jamanetworkopen.2023.8785

Data
Data available: Yes
Data types: Data dictionary
How to access data: lukas.gaffney@childrens.harvard.edu
When available: With publication

Supporting Documents
Document types: None

Additional Information
Who can access the data: Data are publicly available from CMS.
Types of analyses: All are able to access data.
Mechanisms of data availability: All are able to access data.
